# Child with Mongolian spots and dysostosis multiplex

**DOI:** 10.4103/0971-6866.50870

**Published:** 2009

**Authors:** Ketan Prasad Kulkarni, Srinivasa Murthy, Inusha Panigrahi

**Affiliations:** Genetic-Metabolic Unit, Department of Pediatrics, Postgraduate Institute of Medical Education and Research (PGIMER), Chandigarh, India

A 2 year male child, product of a non-consanguineous marriage, presented with recurrent respiratory tract infections since birth, dysmorphic facies and global developmental delay. Excessive increase in head size was noted by parents since the preceding 6 months. There was no history of seizures. The child also had an elder sibling with recurrent respiratory infections and coarse facies who expired at 5 years of age. On examination the child had a normal weight for age (13.8 kg), height for age (85 cm) and occipitofrontal circumference (49 cm). He had coarse dysmorphic facies with depressed nasal bridge, small nose, thick lips, open mouth with protruded tongue and delayed dentition [Figure 1a]. There was pallor, hirsutism and cornea was clear [Figure 1a]. No cherry red spot was detected on fundoscopy. He had extensive Mongolian spots over back [Figure 1b]. He also had distended abdomen, stretched umbilicus, massive hepatosplenomegaly [liver 6 cm (span 11 cm) and spleen 7 cm under costal margins] with left inguinal hernia and knock knees. Investigations revealed anaemia, elevated transaminases and alkaline phosphatase. Urine examination was twice positive for mucopolysaccharides. Skeletal skiagrams revealed inferior beaking of the lumbar vertebrae [Figure 2a], osteopenia, proximal pointing of the metacarpals, bullet shaped phalanges [Figure 2b], thick ribs and calvarium suggestive of dysostosis multiplex. Iduronate-2-sulphatase enzyme assay showed decreased levels. The developmental assessment by Vineland social maturity rating was 40.

**Figure 1a:**
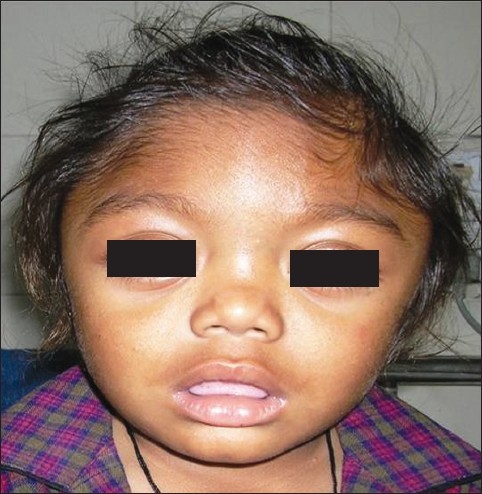
Facial features of the index patient showing coarse dysmorphic facies with depressed nasal bridge, small nose, thick lips and open mouth with protruded tongue

**Figure 1b:**
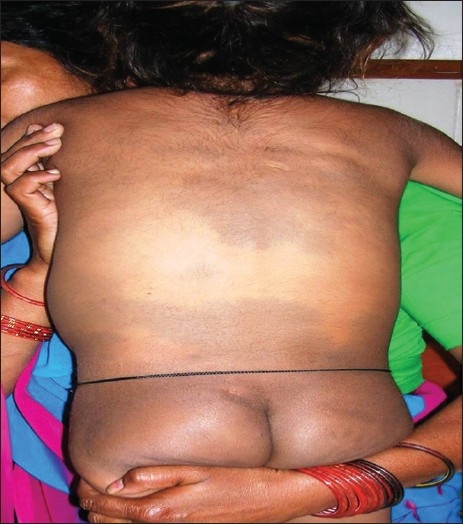
Extensive Mongolian spots over the back

**Figure 2a:**
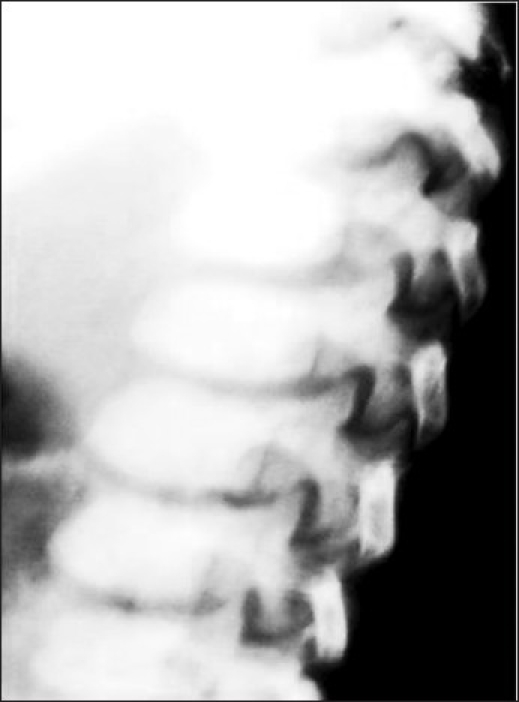
Skiagram of the spine showing inferior beaking of the lumbar vertebrae

**Figure 2b:**
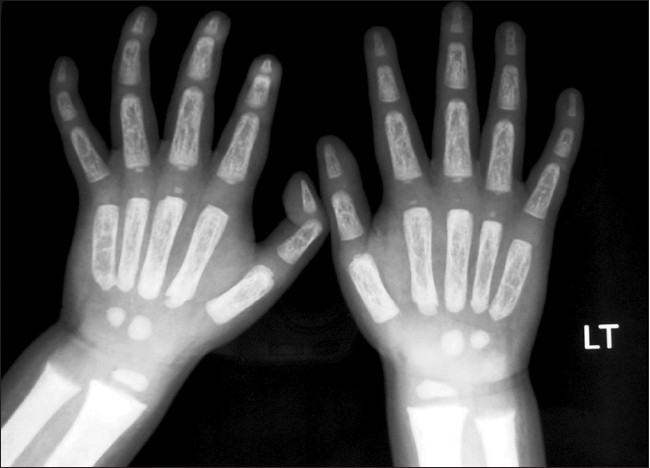
X ray of the hands showing osteopenia, proximal pointing of the metacarpals, bullet shaped phalanges

Thus, in view of characteristic clinical presentation, radiology and enzyme analysis, the child was diagnosed to have Hunter syndrome. Extensive Mongolian spots have been described in patients with Hunter syndrome due to increased melanosomes in the dermis and can aid in early diagnosis.[1] Till recently, the management of this condition was largely supportive but with advent of enzyme replacement therapy[2] and bone marrow transplantation improved outcome is likely.[3]
